# Integrative analysis of triphenyl phosphate: contextual interpretation of bladder cancer cohort

**DOI:** 10.3389/fonc.2023.1260114

**Published:** 2023-10-05

**Authors:** Xiaolei Zhang, Wen Huang, Tao Huang, Jiayi Zhang, Aiming Xu, Yidong Cheng, Chao Qin, Qiang Lu, Zengjun Wang

**Affiliations:** ^1^ Department of Urology, The First Affiliated Hospital of Nanjing Medical University, Nanjing, China; ^2^ Department of Good Clinical Practice (GCP) Office, Nanjing First Hospital, Nanjing Medical University, Nanjing, China

**Keywords:** bladder cancer, triphenyl phosphate, organophosphate ester flame retardants, comparative toxicogenomics databases, bladder cancer progression

## Abstract

In recent years, organophosphate ester flame retardants (OPFRs) have emerged as preferred alternatives to brominated flame retardants (BFRs) in materials such as building supplies, textiles, and furnishings. Simultaneously, a notable surge in bladder cancer incidences has been observed globally, particularly in developed nations, placing it as the 10th most prevalent cancer type. Among the extensive OPFRs, the linkage between triphenyl phosphate (TPP) and bladder cancer remains inadequately investigated. Hence, our study endeavors to elucidate this potential association. We sourced transcriptome profiles and TPP-related data from The Cancer Genome Atlas and Comparative Toxicogenomics databases. Using the ssGSEA algorithm, we established TPP-correlated scores within the bladder cancer cohort. Differentially expressed analysis enabled us to identify key genes in bladder cancer patients. We utilized the LASSO regression analysis, along with univariate and multivariate COX regression analyses to construct a prognostic prediction model. To uncover critical pathways involving key genes, we employed GSEA and GSVA enrichment analyses. Molecular docking analysis was performed to determine the binding capability between TPP and proteins. Our findings reveal that the TPP-centric risk model offers valuable prediction for bladder cancer cohorts. Furthermore, the reliability of this TPP-influenced risk model was verified through ROC curve analysis and survival studies. Intriguingly, TPP exposure appears to bolster the proliferation and invasiveness of bladder cancer cells. This study furnishes new insights into the possible benefits of minimizing TPP exposure for hindering bladder cancer progression.

## Introduction

In recent years, the use of organophosphorus flame retardants (OPFRs) as an alternative to brominated flame retardants is attracting the attention of researchers (1). The flame retardant triphenyl phosphate (TPP) is widely used in consumer products as a plasticizer and flame retardant (2). Although TPP and polybrominated diphenyl ethers (PBDEs) have both been reported as environmental pollutants, the concentrations of TPP are significantly higher in the environment (3). Due to its inability to form chemical bonds with materials, TPP dissolves readily in the environment, allowing it to be detected in a wide range of environmental or biological samples (4). It is primarily released into the environment through volatilization of plastics, manufacturing processes, hydraulic oil spills, and leaching of petroleum products (5). In the environment and biota, TPP is one of the most commonly detected OPFRs. There are generally a few nanograms to several hundred nanograms per liter of surface water with this substance (6). There have been detections of TPP in the microenvironment of our daily lives, including house dust (65-862,000 ng/g) and air (0.00079-220 ng/m^3^). The presence of TPP was also detected in tap water and rice, with levels of 21.27 ng/L and 26.14 ng/g wet weight, respectively (7). There is evidence that TPP is present in the placenta, blood, urine, and breast milk of humans. The environmental problems and health risks associated with TPP are attracting widespread attention, even though it has not been clearly defined as a toxic chemical (8). Recently, many studies focused on the correlation between TPP and cancer. The exposure of TPP could also promote the proliferation and invasive ability of kidney cancer cells (9). Additionally, the TPP has also been regarded as the potential threat to colorectal cancer. Therefore, it is of great importance to evaluate the role of TPP in cancers (10).

Currently, there has been a steady increase in bladder cancer incidence throughout the world, especially in developed countries, and it is the 10th most common cancer worldwide (11). The urothelial cells of the bladder are responsible for 90% of bladder cancer cases, especially in developed nations (12). There is no doubt that environmental and occupational chemicals play a major role in bladder cancer. Tobacco smoke is by far the most common cause (13). Occupational exposure to carcinogens such as aromatic amines, polycyclic aromatic hydrocarbons, and chlorinated hydrocarbons are the second most preventable risk factor for bladder cancer (14). A variety of industrial products containing these compounds are produced, including dyes, paints, metals, rubber, and petroleum (15). As is shown in the previous study, working in “storage and transportation” in the rubber industry was associated with a 253-fold increased risk of death, and “general work” in the rubber industry was associated with a 159-fold increased risk of death (16). Among the other industries associated with an increased risk of bladder cancer are firefighting, hairdressing, and farming with fungicides (17). Despite 2 years of exposure appearing sufficient to increase a person’s risk, the disease often does not manifest itself until decades after exposure, as with tobacco smoke (18).

Recently, with the rapid development of the bioinformatics analysis, more and more relevant researchers have applied the bioinformatics into diagnosis, treatment and prognostic prediction of multiple diseases. In this work, we aim to explore the role of TPP in bladder cancer cohort. The GO and KEGG enrichment analysis was used to explore the potential pathways involved in TPP. The TPP-related prognostic prediction model was constructed to explore the key genes in bladder cancer cohort. The bioinformatics analysis may help to provide insights into bladder cancer etiology, which can also help with early diagnosis and early treatment of bladder cancer.

## Methods

### Collection and organization of data

Interacting genes linked to a range of OPFRs and their metabolites were collected from the Comparative Toxicogenomics Database (CTD, http://ctdbase.org/). Our analysis included 18 distinct OPFRs, such as Phosphoric acid tris(2-methylphenyl) ester and Tributyl phosphate.

### Employing the single-sample gene set enrichment analysis algorithm

To ascertain the extent of OPFR infiltration in patients with bladder cancer, ssGSEA was deployed using the Gene Set Variation Analysis (GSVA) package within R software. Individual gene sets for each OPFR were compiled from the CTD database. Each type of OPFR’s relative abundance was determined through ssGSEA scores, which were normalized between 0 (minimum) and 1 (maximum). A Benjamini-Hochberg corrected p-value less than 0.05 was deemed statistically significant.

### Application of gene ontology and Kyoto Encyclopedia of genes and genomes pathway analysis

The clusterProfiler package within R was applied to analyze genes identified from each module, leveraging Gene Ontology, Kyoto Encyclopedia, and genomic pathway data. Multiple comparison p-value adjustments were conducted using the Benjamini-Hochberg method, with the threshold of statistical significance set at 0.05.

### Construction of a prognostic prediction model based on TPP-related score

The “limma” package in R was harnessed to identify unique genes between two separate TPP-related clusters. The least absolute shrinkage and selection operator (LASSO) analysis was employed to curtail overfitting. Subsequently, LASSO and COX regression analyses were used to devise the prognostic prediction model. Based on the risk model parameters, patient groups were classified into high- and low-risk categories. Survival disparities between these groups were evaluated via a log-rank test, with the risk model’s sensitivity and specificity assessed using ROC curve analysis. Univariate and multivariate independent prognosis analyses were utilized to evaluate the impact of other clinical characteristics on the prognostic value of the risk model. Finally, a nomogram integrating clinical characteristics and a risk model was created, with a calibration curve generated to assess the nomogram’s accuracy.

### Implementation of gene set enrichment analysis and gene set variation analysis

GSEA and GSVA were executed on the dataset to pinpoint pathway genes. We divided the samples into high and low groups based on median signature scores. The GSEA V4.1.0 software was used for GSEA, employing a background gene set of c2.cp.kegg.v7.2.symbols.gmt. For GSVA, the “GSVA” R package was utilized.

### Conducting molecular docking

Molecular docking procedures were carried out via VLife molecular docking software version 4.6.10, utilizing PDB obtained from the RCSB PDB-101 Protein Crystal Database (PDB, http://www.rcsb.org/pdb/). The resultant docked complexes were examined in the Interaction segment of the software.

### Culture of bladder cancer cells

T24 bladder cancer cells were maintained in DMEM, complemented with 15% fetal bovine serum, 10% fetal calf serum, and 1% penicillin-streptomycin.

### Application of CCK8 assay

We employed the CCK8 kit (Dojindo, Shanghai, China) for measurements. Each well of a 96-well plate was seeded with 2000 cells, with 10 liters of CCK8 added to each well. A humidifier was used to incubate them at 37°C for 1.5 hours with 5% CO2.

### Performance of transwell assay

The invasive potential of bladder cancer cells was evaluated using Transwell chambers, in accordance with the manufacturer’s guidelines. Transwell chambers containing 24 wells were filled with DMEM medium having 0.5x 106 cells per ml. To assess the invasive capability of cancer cells, Extra Matrigel was injected into the upper chamber. After 24 hours in a humidified incubator containing 5% CO, the membrane was treated with Methanol for five minutes and then dried at room temperature for 30 minutes. Following this, the membrane was stained with crystal violet at room temperature for 20 minutes before examination under a light microscope.

### Execution of statistical analysis

All statistical analyses in this study were performed using R software. A P-value less than 0.05 was considered statistically significant, unless otherwise specified.

### Discovery of critical genes linked with OPFRs and establishment of an OPFRs-related score system

**Figure 1:**
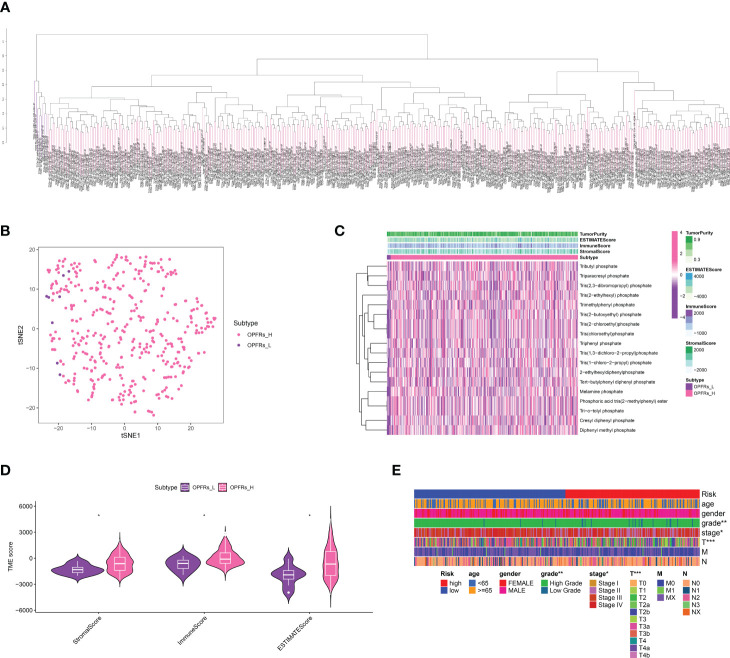
**(A)** The ssGSEA algorithm revealed the OPFRs-related scores in bladder cancer cohort; **(B)** The bladder cancer cohort was divided into OPFRs-low and OPFRs-high group based on the ssGSEA algorithm; **(C)** The heatmap demonstrated the specifical OPFRs-related score in bladder cancer patients; **(D)** The correlation between TME and OPFRs-related score; **(E)** The heatmap revealed the correlation between clinical features and TPP-related score. “*” means P<=0.05, “**” means P<=0.01, “***” means P<=0.001.

Initially, we extracted genes interacting with 18 different types of OPFRs and their metabolites from the CTD database. Utilizing the ssGSEA algorithm, we quantified an OPFRs-related score for each individual in the bladder cancer cohort, which led to the categorization of the cohort into high and low OPFRs groups (**Figures 1A, B**). These scores were illustrated via a heatmap (**Figure 1C**), and we undertook a correlation study to explore the association between these scores and the tumor microenvironment (TME) (**Figure 1D**). The findings suggested a direct correlation between elevated OPFRs-related scores and immune, stromal, and ESTIMATE scores.

### Evaluation of relationships between TPP-related scores and clinical attributes, immune cells, and immune checkpoint-related genes

**Figure 2:**
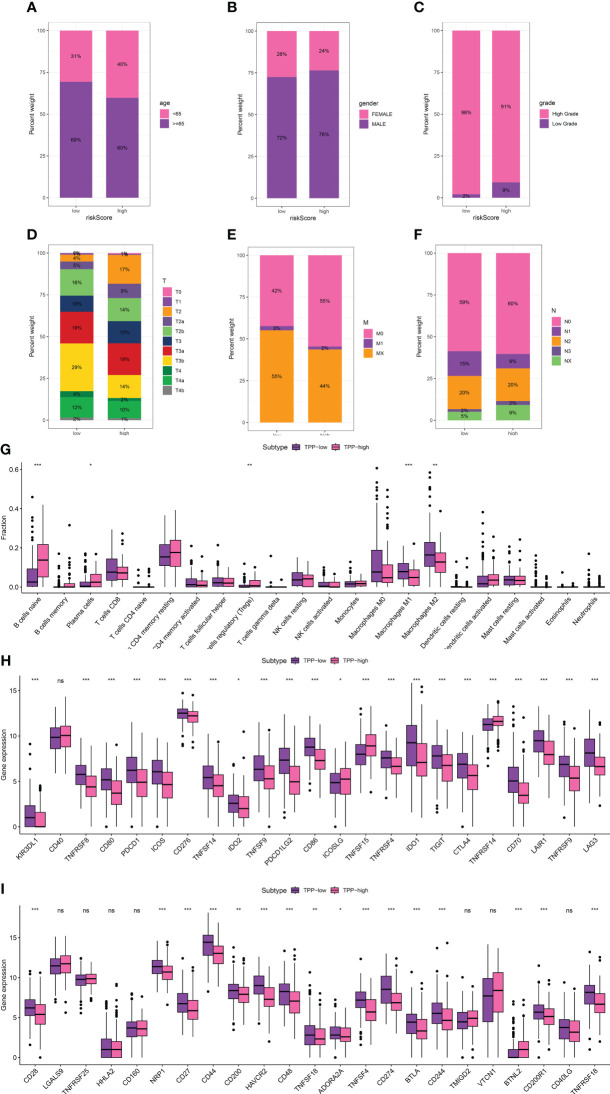
The correlation between TPP-related score and age **(A)**, gender **(B)**, grade **(C)**, T stage **(D)**, M stage **(E)** and N stage **(F**, **G)**. The correlation between immune-related cells and TPP-related scores; **(H, I)** The correlation between expression level of immune checkpoint-related genes and TPP-related scores. “*” means P<=0.05, “**” means P<=0.01, “***” means P<=0.001. ns, not statistically significant.

We delved into the association between TPP-related scores and clinical attributes, revealing a significant correlation with patient age, gender, and disease stage (**Figure 1E**). Reduced TPP-related scores were linked with increased age (over 65), lower grade and T/M stages, whereas elevated TPP-related scores were associated with male patients and a higher N stage (**Figures 2A–F**). Moreover, heightened TPP-related scores coincided with enhanced infiltration of naive B cells, plasma cells, regulatory T cells, and M1/M2 macrophages (**Figure 2G**). An association analysis between the expression levels of immune checkpoint-related genes and TPP-related scores demonstrated that elevated expression of these genes was associated with diminished TPP-related scores (**Figures 1H, I**).

### Establishment of the TPP-related prognostic prediction model in the bladder cancer cohort

**Figure 3:**
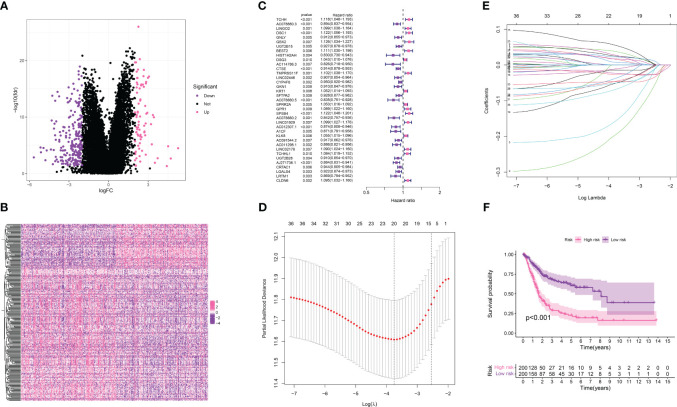
**(A)** The differentially expressed analysis between TPP-low and TPP-high groups; **(B)** The heatmap demonstrated the expression level of differentially expressed genes in bladder cancer patients; **(C)** The univariate revealed the prognosis-related genes in bladder cancer cohort; **(D, E)** The LASSO regression analysis; **(F)** The survival analysis between low- and high-risk groups.

In our endeavor to pinpoint the genes crucial in the bladder cancer cohort, we performed a differential expression analysis between the TPP-low and TPP-high groups. This identified 232 down-regulated genes and 79 up-regulated genes, considered to be key within TPP (**Figures 3A, B**). The heatmap showcased the expression level of these key genes. These 311 genes then became the subjects of further analysis. A univariate COX regression analysis isolated prognosis-related genes (**Figure 3C**), identifying 37 such genes (**Figures 3D, E**). Subsequent LASSO regression analysis refined this list, with a multivariate COX regression analysis used to build the risk model. Each patient with bladder cancer was assigned a risk score as per the following formula: Risk score = TCHH * 0.0588545960366445 + LINGO2 * 0.0571622902543735 + GNLY * -0.258747524466991 + HIST1H2AH * -0.249755464196645 + TMPRSS11F * 0.0939659133443133 + AC078880.5 * -0.113301124037407 + A1CF * -0.0726391262458251 + AC091544.2 * -0.087891281461466 + AC011298.1 * -0.0715856458719754 + AJ271736.1 * -0.0572227894080378 + LRTM1 *-0.102138079651338. The survival analysis indicated that higher risk scores correlated with poorer overall survival (OS) in bladder cancer patients (**Figure 3F**).

### Evaluation of the risk model’s function and construction of a prognostic-related nomogram in bladder cancer cohorts

**Figure 4:**
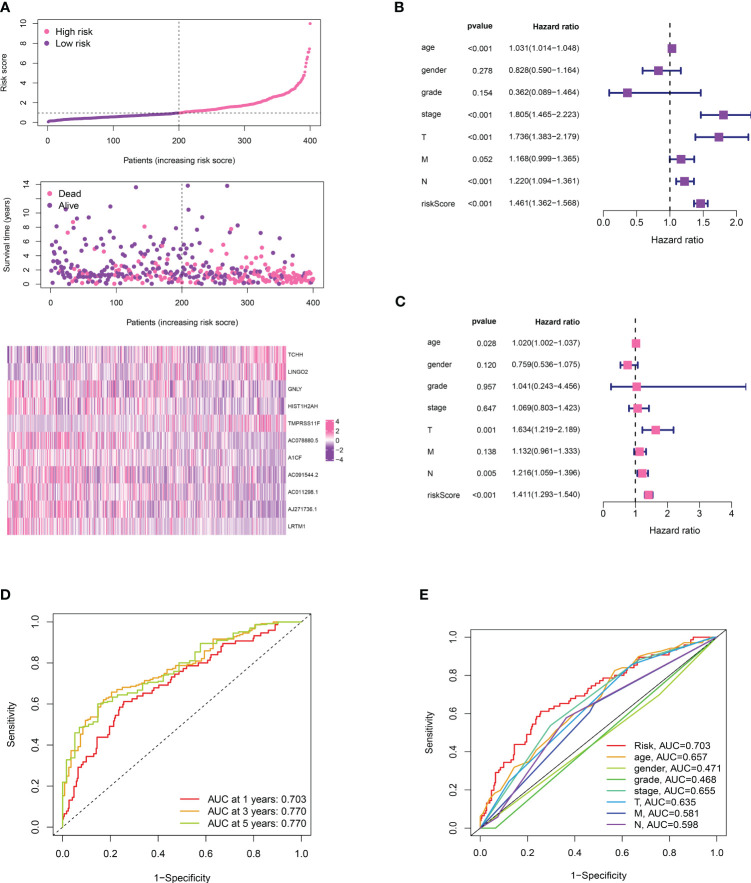
**(A)** The risk plot revealed the survival status of bladder cancer patients; **(B)** The univariate independent prognosis analysis; **(C)** The multivariate independent prognosis analysis; **(D)** The time-dependent ROC curve revealed the AUC score of 1-year, 3 year and 5-year in bladder cancer cohort; **(E)** The ROC curve revealed the AUC score of risk score and clinical features.

**Figure 5:**
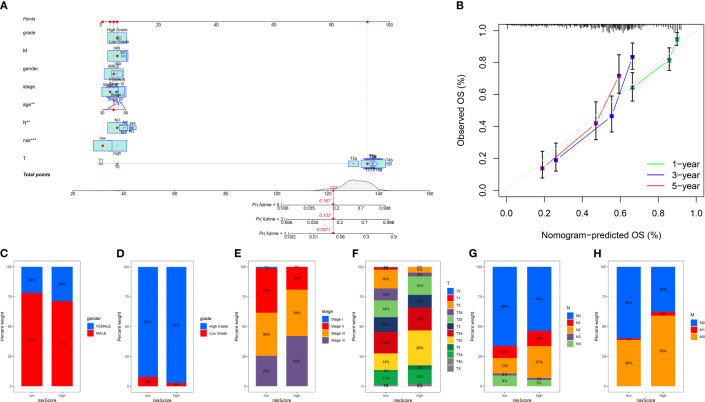
**(A)** The nomogram based on risk score and clinical features; **(B)** The calibration curve shows the predictive value of nomogram; The correlation analysis between risk score and gender **(C)**, grade **(D)**, stage **(E)**, T stage **(F)**, N stage **(G)** and M stage **(H)**. “**” means P<=0.01, “***” means P<=0.001.

We divided the bladder cancer cohort into low- and high-risk groups based on the median risk scores derived from the TPP-based prognostic model (**Figure 4A**). Higher risk was linked to poorer overall survival. Independent prognosis analysis identified age, disease stage, and risk scores as significant independent risk factors for bladder cancer (**Figures 4B, C**). The time-dependent ROC curve revealed AUC scores of 0.703, 0.770, and 0.772 for 1-year, 3-year, and 5-year periods, respectively (**Figure 4D**). The clinical ROC curve showed that the predictive value for risk score was superior to that of clinical features (**Figure 4E**). Our constructed nomogram demonstrated robust predictive value for bladder cancer patients, with our analysis suggesting a link between higher risk scores and advanced clinical features (**Figure 5**).

### Probing the binding affinity between TPP and proteins encoded by risk model-associated genes

**Figure 6:**
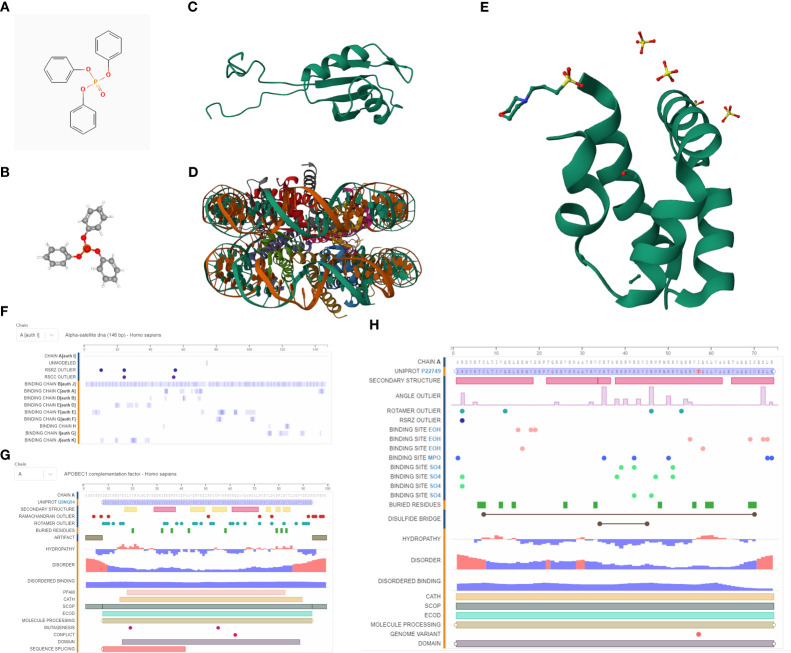
**(A)** The 2D structure of TPP; **(B)** The 3D structure of TPP; **(C)** The 3D structure of A1CF protein; **(D)** The 3D structure of HIST1H2AH protein; **(E)** The 3D structure of GNLY protein; **(F)** The sequences of HIST1H2AH analyzed by NMR spectroscopy; **(G)** The sequences of A1CF analyzed by NMR spectroscopy; **(H)** The sequences of GNLY analyzed by NMR spectroscopy.

**Figure 7:**
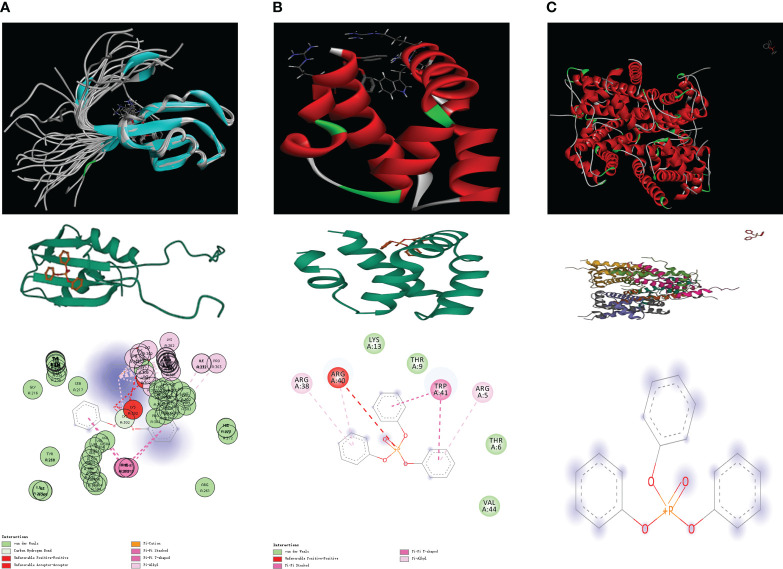
**(A)** The molecular docking between TPP and A1CF; **(B)** The molecular docking between TPP and GNLY; **(C)** The molecular docking between TPP and HIST1H2AH.

We procured the structures of TPP and several pivotal proteins using the PubChem and RCSB databases. A molecular docking examination between TPP and proteins A1CF, GNLY, and HIST1H2AH revealed differential binding affinities. A1CF exhibited robust binding with TPP, GNLY demonstrated intermediate binding, while HIST1H2AH did not bind (**Figures 6**, **7**).

### Uncovering potential pathways of A1CF and TPP-associated genes in the bladder cancer cohort

**Figure 8:**
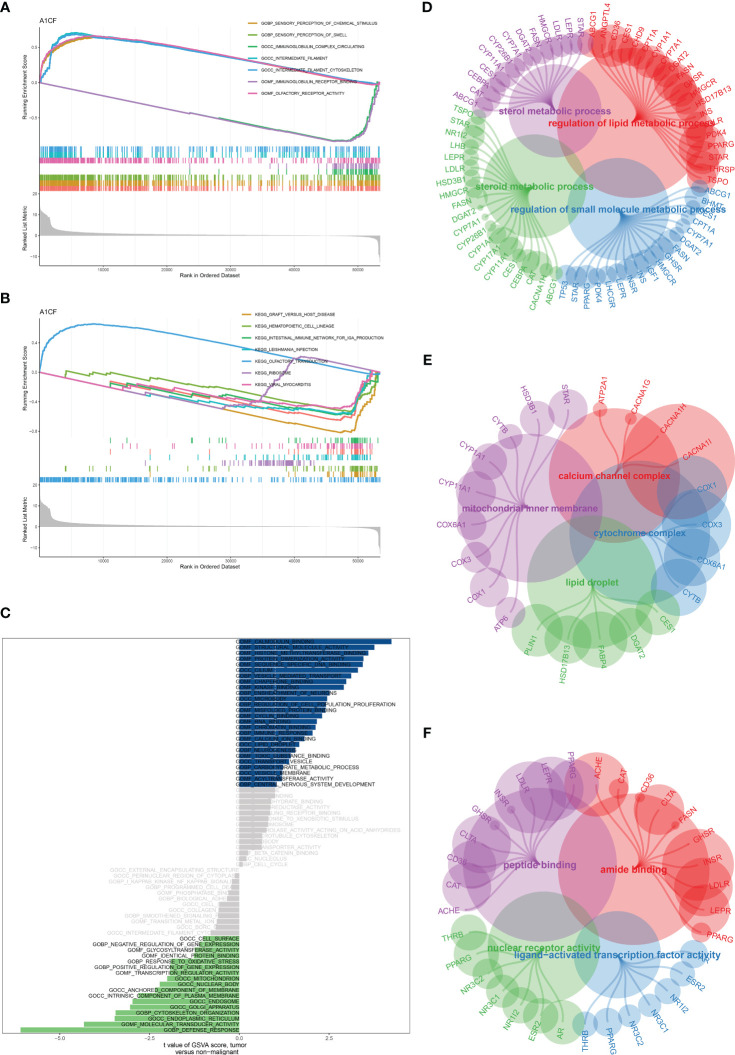
**(A)** The GSEA of A1CF based on GO terms; **(B)** The GSEA of A1CF based on KEGG terms; **(C)** The GSVA of A1CF in bladder cancer cohort; **(D)** The GO BP enrichment analysis based on TPP-related genes; **(E)** The GO CC enrichment analysis based on TPP-related genes; **(F)** The GO MF enrichment analysis based on TPP-related genes.

Our findings indicated a strong correlation between the gene A1CF and TPP exposure in bladder cancer cohorts. To discover potential pathways associated with A1CF, we conducted Gene Set Enrichment Analysis (GSEA) and Gene Set Variation Analysis (GSVA). The GSEA revealed that the most significantly enriched Gene Ontology (GO) terms included chemical sensory perception, olfaction, immunoglobulin complex circulation, intermediate filament, and intermediate filament cytoskeleton (**Figure 8A**). Notably, the KEGG pathways most enriched encompassed graft-versus-host disease, hematopoietic cell lineage, IgA production within the intestinal immune network, and olfactory transduction (**Figure 8B**). In the GSVA, pathways including defense response, molecular transducer activity, calmodulin binding, structural molecule activity, and positive gene expression regulation were prominent (**Figure 8C**). Subsequent GO enrichment analysis for TPP-associated genes in the Biological Process (BP) category revealed enrichment of pathways related to steroid metabolism, regulation of lipid metabolism, regulation of small molecule metabolism, and sterol metabolism (**Figure 8D**). In the Cellular Component (CC) category, the cytochrome complex, lipid droplets, mitochondrial inner membrane, and calcium channel complex were most enriched (**Figure 8E**). In the Molecular Function (MF) category, pathways associated with nuclear receptor activity, ligand-activated transcription factor activity, amide binding, and peptide binding were deemed highly correlated (**Figure 8F**). These outcomes provide a broad understanding of the potential molecular pathways impacted by TPP in bladder cancer cells.

### The exposure of TPP could promote the cell invasion and proliferation ability of bladder cancer cells

**Figure 9:**
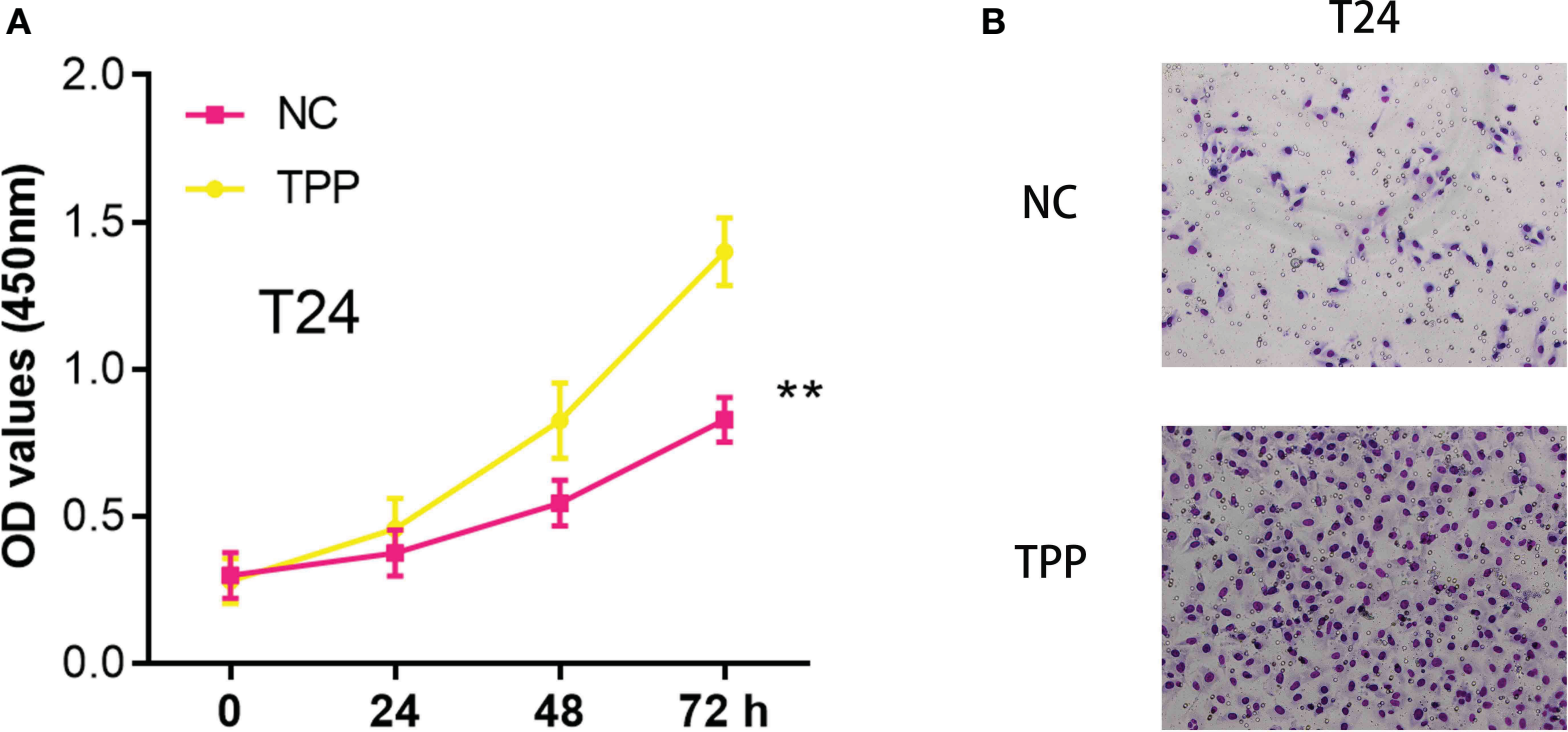
**(A)** The CCK8 assay in T24 cells with the mock exposure and none exposure of TPP at the concentration of 10^-6^ M; **(B)** The Transwell assay in T24 cells with the mock exposure and none exposure of TPP at the concentration of 10^-6^ M. “**” means P<=0.01.

In our investigation, we found that exposure to Triphenyl Phosphate (TPP) significantly enhanced the invasiveness and proliferative capacity of bladder cancer cells. A series of *in vitro* experiments were conducted where the cells were treated with TPP. Subsequent CCK8 assays demonstrated a dose-dependent increase in cell proliferation rates upon TPP exposure (**Figure 9A**). Furthermore, invasion assays showed a marked enhancement in the invasive potential of these cells under similar treatment conditions (**Figure 9B**).

## Discussion

In 2018, OPFRs accounted for 30% of the global flame-retardant market as an alternative to brominated flame retardants (8). There are now multiple pathways for humans to become exposed to OPFRs due to the increased use of these particles in household dusts and food workers (19). OPFR is primarily transmitted through inhalation or dust inhalation in young children who spend most of their time at home, while food inhalation is predominant in adolescents and adults (20). The modern world is therefore filled with humans exposed to OPFR. The TPP aryl-OPFR is widely used in a variety of products such as baby products, home furnishings, electronic equipment, and building materials (21). As an additive flame retardant, TPP does not form chemical bonds with materials, and is therefore easily released into the environment (22). In recent years, TPP has been discovered to be closely associated with many cancers, such as prostate cancer and colorectal cancer. However, no study focused on the correlation between TPP and bladder cancer. As one of the most common developed tumors in the urinary system, bladder cancer is considered to be closely correlated to the environmental exposure (23). In addition, suspected/established occupational bladder carcinogens include 2-naphthylamine, 4-aminobiphenyl, toluene, 4,4’-methylenebis(2-chloroaniline), metalworking fluids, polyaromatic hydrocarbons and vinyl chloride (24). In this work, we aim to explore the correlation between TPP and bladder cancer. First of all, the interactive genes of multiple OPFRs and metabolites of OPFRs were downloaded from the CTD database. In order to obtain the OPFRs-related scores in bladder cancer cohort, we then performed the ssGSEA algorithm. Each bladder cancer patient was evaluated with the OPFRs-related score. The clinical-related analysis revealed that the OPFRs-related score is closely correlated with the clinical-related features, such as grade, stage and T stage. Also, many immune-related cells and expression level of immune-related genes were also closely correlated with OPFRs-related score. As is shown by the former study, the exposure of the TPP could improve the proliferation and invasive ability of prostate cancer cells, which may indicate the toxicity of TPP in prostate cancer patients (9). In addition, multiple studies have evaluated the potential correlation between TPP and thyroid cancer (25). Also, according to a case-control study conducted in eastern China, OPFR exposure increases thyroid cancer risk (26).

Subsequently, we performed the differentially expressed analysis to explore the key genes involved in TPP. On the basis of the TPP-related differentially expressed genes, we then construct the risk model by using COX regression analysis and LASSO regression analysis. The survival analysis and ROC curve revealed that the TPP-related risk model is closely correlated with the prognosis of bladder cancer patients. In addition, the risk model showed great predictive value for bladder cancer patients. In recent years, multiple studies have applied the bioinformatics analysis to evaluate the correlation between environmental toxicity and cancers. As is shown in the previous study, phthalates have been associated with potential negative effects on prostate cancer, and a pharmacological method has been proposed to predict harmful effects (27). In addition, a former study also discovered that multiple water pollutants were considered to be closely associated with the human diseases, such as colon tumors, breast tumors, hepatitis B, bladder cancer, and human cytomegalovirus infection (28). Also, a bioinformatics analysis revealed that perfluorinated compounds may be the potential threaten to bladder cancer patients (29). Therefore, in the future, more study should focus on the correlation between environmental exposure and cancers.

Next, by performing the molecular docking, we discovered that A1CF may be a key biomarker for TPP-induced bladder cancer patients. Apobec-1 complementation factor (A1CF) has been identified as a complement factor of apolipoprotein-B messenger RNA editing (30). According to previous studies, the RNA-binding protein A1CF upregulated DKK1 expression and inhibited Wnt/β-catenin signaling (31). Furthermore, another study demonstrated that A1CF promoted breast cancer cell proliferation and migration and inhibited apoptosis (32). In this work, the molecular docking analysis showed strong binding ability between TPP and A1CF, such as van der waals, pi-cation, carbon hydrogen bond and pi-pi stacked.

Finally, in order to explore the enriched pathways of TPP-related genes and A1CF in bladder cancer cohort, we then performed the GSEA, GSVA and GO enrichment analysis. The results demonstrated that some immune-related pathways and lipid metabolism-related pathways were significantly enriched. A proteomic analysis revealed that related proteins related to apoptosis, oxidative stress, metabolism, and membrane structure were affected (18). Additionally, metabolic pathways such as glycolysis, citric acid cycle, oxidative phosphorylation, lipid and protein metabolism were significantly disrupted (33). Also, the cell proliferation and invasive assays demonstrated that the exposure of TPP could promote the cell proliferation and invasive ability of bladder cancer cells. The implication of such results is profound as it underscores the potential risks associated with TPP exposure and its role in bladder cancer progression. Further substantiating this, our invasion assays revealed a stark increase in cell invasive capacity following TPP treatment. This suggests that TPP might facilitate cancer metastasis, a critical aspect of cancer progression and a leading cause of cancer mortality. However, while our findings paint a concerning picture of the possible implications of TPP exposure, it is necessary to mention that our research, like any, has limitations. The results obtained are from *in vitro* experiments, which, though valuable, do not fully represent the complexity of human physiology. Future studies involving *in vivo* models and clinical investigations would provide a more comprehensive understanding of the potential carcinogenic role of TPP. Also, further molecular and mechanistic studies are required to decipher the exact pathways by which TPP induces these changes in bladder cancer cells.

However, some limitations are also involved in the bioinformatics analysis. Firstly, bioinformatics analysis is heavily reliant on the quality of the initial data. Poor quality or incorrectly annotated data can lead to inaccurate results. In addition, the complex nature of biological systems often means that bioinformatics analyses can oversimplify these systems, which may result in inaccurate modeling or predictions. Besides, algorithms used in bioinformatics are often based on certain assumptions about biological data, which might not always hold true. This can affect the accuracy of the analysis.

In total, in this work, we successfully discovered the correlation between TPP and bladder cancer. A1CF may be considered as the key biomarker for the TPP-induced bladder cancer. Our analysis provided great directions for the future analysis.

## Data availability statement

The original contributions presented in the study are included in the article/supplementary material. Further inquiries can be directed to the corresponding authors.

## Author contributions

XZ: Methodology, Writing – original draft. WH: Software, Writing – original draft. TH: Investigation, Writing – review & editing. JZ: Conceptualization, Writing – original draft. AX: Software, Writing – original draft. YC: Software, Writing – original draft. CQ: Software, Writing – original draft. QL: Investigation, Writing – original draft. ZW: Software, Writing – original draft.
